# Trachelogenin alleviates osteoarthritis by inhibiting osteoclastogenesis and enhancing chondrocyte survival

**DOI:** 10.1186/s13020-024-00909-x

**Published:** 2024-03-01

**Authors:** Tao Jiang, Jiahui Zhang, Beite Ruan, Xiaobing Xi, Zhuo Yang, Jianmin Liu, Hongyan Zhao, Xing Xu, Min Jiang

**Affiliations:** 1 Department of Endocrine and Metabolic Diseases, Shanghai Institute of Endocrine and Metabolic Diseases, Ruijin Hospital, Shanghai Jiao Tong University School of Medicine, Shanghai, China; 2 Shanghai National Clinical Research Center for Metabolic Diseases, Key Laboratory for Endocrine and Metabolic Diseases of the National Health Commission of the PR China, Shanghai Key Laboratory for Endocrine Tumor, State Key Laboratory of Medical Genomics, Ruijin Hospital, Shanghai Jiao Tong University School of Medicine, Shanghai, China; 3 Department of Orthopaedics, Shanghai Key Laboratory for Prevention and Treatment of Bone and Joint Diseases, Shanghai Institute of Traumatology and Orthopaedics, Ruijin Hospital, Shanghai Jiao Tong University School of Medicine, Shanghai, China; 4 The Seventh People’s Hospital of Shanghai University of Traditional Chinese Medicine, Shanghai, China; 5 Chemical Biology Core Facility, Shanghai Institute of Biochemistry and Cell Biology, Center for Excellence in Molecular Cell Science, Chinese Academy of Sciences; University of Chinese Academy of Sciences, Shanghai, China

**Keywords:** Osteoarthritis, Trachelogenin, Subchondral bone, Chondrocyte, Rap1, Glycolysis

## Abstract

**Background:**

Osteoarthritis (OA) is a prevalent global health concern associated with the loss of articular cartilage and subchondral bone. The lack of disease-modifying drugs for OA necessitates the exploration of novel therapeutic options. Our previous study has demonstrated that traditional Chinese medical herb *Trachelospermum jasminoides* (Lindl.) Lem. extract suppressed osteoclastogenesis and identified trachelogenin (TCG) as a representative compound. Here, we delved into TCG’s potential to alleviate OA.

**Methods:**

We initially validated the in vivo efficacy of TCG in alleviating OA using a rat OA model. Subsequently, we isolated primary bone marrow-derived macrophages in vitro to investigate TCG's impact on osteoclastogenesis. We further employed a small molecule pull-down assay to verify TCG's binding target within osteoclasts. Finally, we isolated primary mouse chondrocytes in vitro to study TCG's regulatory effects and mechanisms on chondrocyte survival.

**Results:**

TCG preserved subchondral bone integrity and protected articular cartilage in a rat OA model. Subsequently, in vitro experiments unveiled TCG's capability to inhibit osteoclastogenesis and function through binding to Ras association proximate 1 (Rap1) and inhibiting its activation. Further study demonstrated that TCG inhibited Rap1/integrin αvβ3/c-Src/Pyk2 signaling cascade, and consequently led to failed F-actin ring formation. Besides, TCG promoted the proliferation of mouse primary chondrocytes while suppressing apoptosis in vitro. This is attributed to TCG's ability to upregulate HIF1α, thereby promoting glycolysis.

**Conclusion:**

TCG exerted inhibitory effects on osteoclastogenesis through binding to Rap1 and inhibiting Rap1 activation, consequently preventing subchondral bone loss. Moreover, TCG enhanced chondrocyte survival by upregulating HIF1α and promoting glycolysis. These dual mechanisms collectively provide a novel approach to prevented against cartilage degradation.

**Supplementary Information:**

The online version contains supplementary material available at 10.1186/s13020-024-00909-x.

## Introduction

Osteoarthritis (OA) is the most common degenerative joint disease, causing severe pain and permanent damage to the joints, affecting approximately 500 million adults worldwide [1]. OA carried a great economic burden, with average annual cost per individual estimated to range from $700 to $15,600 (in terms of 2019 USD) in different regions worldwide [2]. Currently, steroidal or nonsteroidal anti-inflammatory drugs (NSAIDs) are available for OA, providing palliative symptomatic relief by reducing pain and inflammation [3]. However, the notable gastrointestinal side effects of these drugs have restricted their clinical utilization [4].

In recent years, research efforts have been devoted to disease-modifying OA drugs (DMOADs) aimed at intervening in the pathophysiology of OA and mitigating structural deterioration to prevent long-term disability [5]. Unfortunately, to the best of our knowledge, there has been limited progress in the development of DMOADs so far. For example, MIV-711, an oral cathepsin K inhibitor, did not show pain improvements after 26 weeks of administration [6]. Lorecivivint improved patient pain in phase II trials but did not meet the primary endpoint [7]. This might partly attribute to the absence of a singular, unified cellular or molecular cascade associated with the early OA process, as well as the substantial heterogeneity of the disease. Therefore, development of effective DMOADs remains a challenge and highlights the need for alternative therapeutic strategies.

Historically, the pathogenesis of OA has been associated with a simple progressive "wear and tear" process. Cartilage degeneration is a prominent feature of OA. Currently, attention to OA has now broadened to encompass its multifaceted nature, which includes factors such as osteophyte growth, subchondral bone remodeling and synovial inflammation. Among these, the subchondral bone is emerging as a promising therapeutic target. In fact, articular cartilage-subchondral bone has been identified as a functional complex that play a complementary role in dynamic load bearing [8]. During OA progress, abnormal mechanical stress triggers increased osteoclast-mediated subchondral bone remodeling, causing bone cyst and microfractures [9]. Compromised subchondral bone subsequently transmits elevated loads to the overlying cartilage, and exacerbated its deterioration. As a novel therapeutic strategy, addressing this abnormal bone remodeling by targeting osteoclasts could potentially improve early OA outcomes by maintaining the integrity of subchondral bone and providing solid support for the articular cartilage. This was confirmed by a study reporting a 26% reduction in joint replacement surgery rates among bisphosphonate drug users [10].

Considering the substantial heterogeneity of OA, natural products that offer multi-target interventions by acting through various pathways and creating synergistic effects are being regarded as potential candidates for innovative treatment approaches [11]. *Trachelospermum jasminoides* (Lindl.) Lem. (*T. jasminoides*) is a traditional Chinese medicinal herb renowned for its alleviating effects on joint discomfort [12]. Notably, the content of tracheloside in *T. jasminoides* is specifically defined as a quality standard in the *Chinese Pharmacopoeia (2020 Edition)*. Pharmacokinetic study showed that the glycosidic bond of tracheloside was cleaved in mice stomach and converted to trachelogenin (TCG), indicating TCG as the bioactive compound in vivo [13]. Nevertheless, the potential role of TCG in addressing OA remains unexplored.

Our preliminary study has unveiled the anti-osteoclastogenesis effects of *T. jasminoides* extract [14]. Considering the vital role of osteoclasts in the subchondral bone remodeling during the pathogenesis of OA, we hypothesized that TCG, as a representative compound of *T. jasminoides*, could potentially alleviate OA by modulating osteoclasts in subchondral bone. Therefore, in this study, we initially established a rat OA model induced by joint instability and explored the pharmacological effects of TCG in OA prevention. Moreover, we further aimed to elucidate the mechanistic insights into TCG's regulation on subchondral bone and articular cartilage by employing in vitro methods including RNA sequencing and proteomics analysis.

## Materials and methods

### Animal study and reagent

Male Sprague Dawley rats weighing 260–280 g were sourced from Zhejiang Vital River Laboratory Animal Technology. These rats were accommodated in a specific pathogen-free-grade animal cabin, ensuring unrestricted access to food and water. The housing conditions were meticulously controlled, maintaining a 12-h light/dark cycle, 60–80% humidity, and a temperature of 22 ± 1 °C. After acclimatization, OA was induced using the destabilization of the medial meniscus (DMM) method [15]. After anesthesia and disinfection, a surgical incision in the medial parapatellar skin revealed the knee joint. Then, the medial meniscotibial ligament was carefully transected. The joint was closed, and the rats were allowed to recover. Starting at the 7th day after surgery, intra-articular injections of either 100 µg or 200 µg TCG (CAS: 34209-69-3, Purity ≥ 98%, Tianzhi Biotechnology, Wuhan, China) or an equivalent volume of vehicle were administered twice a week for 8 weeks. The rats were sacrificed for further study. The animal experiments were conducted in compliance with the Guide for the Care and Use of Laboratory Animals (National Institutes of Health publication 85–23, revised 1996) and were approved by the Shanghai Jiao Tong University School of Medicine Animal Study Committee (2018-0027).

### Histological analysis

The isolated joints were fixed in 4% paraformaldehyde (PFA) for 7 days, then decalcified, paraffin embedded, and sliced. The sections underwent staining using safranin O and fast green (S–O/FG), hematoxylin–eosin (HE), and immunohistochemical staining with anti-Collagen II antibody (Col2a1). OARSI cartilage histopathology assessment system was applied to evaluate the extent of articular cartilage damage [16]. The overall OARSI score, ranging from 0 to 24, was determined by multiplying OA grade (ranging from 0 to 6) and OA stage (ranging from 0 to 4).

### Von Frey test

To assess pain sensitivity in rats, Von Frey hairs (Semmes–Weinstein Monofilaments, Galesville, USA) were used as previously reported [17]. Rats were acclimated to the testing environment for 20 min. Filaments with different forces were applied to the hind paw, starting from 0.4 g. Responses were recorded as "O" for negative and "X" for positive. The formula for calculating 50% paw withdrawal threshold (PWT) is “50% PWT = 10^[Xf+kδ]^/10^4”^, in which Xf represent the value (in log units) of the last filament used, k is related to the response pattern (modified from Dixon), and δ is a constant (in log units) determined by standard deviation (SD) of serial force.

### Micro-CT analysis

The isolated joints were fixed in 4% PFA for 7 days. Subsequently, a high-resolution micro-CT scanner (SkyScan-1176, Bruker, Belgium) was used to conduct quantitative analysis of knee joints. Morphometric parameters were calculated through 3D analysis using CTAn software (Bruker, Belgium). The visual 3D reconstruction was created by CTvox software (Bruker, Belgium). More importantly, all analyses were carried out in a blinded manner.

### Isolation of mouse primary cells and reagent treatment

Bone marrow-derived macrophages (BMMs) were isolated from male C57/BL6 mice at the age of 4 to 6 weeks by collecting bone marrow cells from the femurs and tibias as previous reported [18]. The cells were incubated with α-modified minimal essential medium (α-MEM) complete medium with 1% penicillin/streptomycin (PS) and 10% fetal bovine serum (FBS). BMMs were induced into osteoclasts under the stimulation of 30 ng/mL macrophage-stimulating factor (M-CSF) and 50 ng/mL nuclear factor kappa-B ligand (RANKL).

Primary mouse chondrocytes (PMCs) were isolated from neonatal C57BL/6 mice knee cartilage within 5 days after birth as previous reported [19]. The harvested cells were incubated in Dulbecco’s Modified Eagle Medium/Nutrient Mixture F-12 (DMEM F12) with 1% PS and 10% FBS at 37 °C in a normal oxygen incubator with 20% oxygen or in a low oxygen incubator with 1% oxygen (Esco Lifesciences, Singapore).

8-pCPT-2′-O-Me-cAMP-AM (CAS: 1152197-23-3) used in this article to activate Ras association proximate 1, Rap1) were purchased from APExBIO (Huston, USA). 2-Deoxy-D-glucose (CAS: 154-17-6, 2-DG) and LW6 (CAS: 934593-90-5) used to treat PMCs were obtained from MedChemExpress (Monmouth Junction, USA).

### TRAP staining and Phalloidin staining

BMMs were induced to form osteoclasts while being treated with indicated doses of biotin, TCG, or TCG-biotin. Osteoclasts were stained using the leukocyte acid phosphatase (TRAP) staining kit (Sigma-Aldrich Inc., St. Louis, USA) following the manufacturer's instructions. Under the microscope, cells exhibiting a burgundy color and possessing multiple nuclei (three or more) were considered as osteoclasts for inclusion in the analysis. To visualize F-actin, the cells were incubated with rhodamine phalloidin (2 units/mL) for 30 min away from light. 4,6-diamidino-2-phenylindole (DAPI, 1 μg/mL) was used to stain nuclei. The F-actin ring was observed as red ring under fluorescence microscope.

### Bone resorption assay

To investigate the impact of TCG on the osteoclast bone resorption function, BMMs were induced into osteoclasts in a collagen-coated dish. Subsequently, the cells were gently transferred onto bone slices and treated with a specified dose of TCG for 48 h. As previously described, bone resorption pits were assessed using a confocal laser scanning microscope [20].

### CCK-8 assay

BMMs and PMCs were exposed to indicated doses of TCG for a specified period of time. Following treatment with CCK-8 reagent, cell viability was assessed by measuring the optical density at 450 nm using the Infinite F200 PRO absorbance microplate reader. The calculated cell viability was normalized to the control.

### qRT-PCR analysis

RNA extraction reagent (Vazyme Biotech, Nanjing, China) was used to extract the total RNA from the sample of interest. The concentration and purity of RNA were measured using a NanoDrop spectrophotometer. Then, PrimeScript Reverse TranscriptMasterMix (TaKaRa, Otsu, Japan) was employed to conduct reverse transcription. Subsequently, QuantStudio Real-Time PCR system (Thermo Fisher, Waltham, USA) was utilized to perform qRT-PCR. Details of Primer sequences are listed in Additional file 2: Table S1. The relative mRNA expression was assessed using the ΔΔCT method.

### Western blot and immunofluorescence assay

Total protein lysates were collected. Lysates were then subjected to SDS-PAGE and subsequently transferred to polyvinylidene fluoride membranes. After 1-h of membrane blocking, the membrane was then incubated overnight at 4 °C with primary antibodies (Additional file 2: Table S2). Next day, after a 1-h incubation with secondary antibodies at room temperature, chemiluminescence reagents and eBlot Touch Imager (eBlot, Shanghai, China) were utilized to detect protein bands.

For immunofluorescence assays, cells were fixed, permeabilized, and blocked. Subsequently, the cells were incubated overnight at 4 °C with p65 antibodies (1:400 dilution). Following this, cells were incubated for 1 h at room temperature with Alexa Fluor 488-conjugated antibodies. Images were captured using a Zeiss LSM880 confocal microscope.

### RNA sequencing analysis

The total RNA from each sample was prepared and subjected to quality control using the Agilent bioanalyzer 2100 (Agilent, Santa Clara, USA). Subsequently, the RNA is converted into a cDNA library using NEBNext Ultra RNA Library Prep Kit (Illumina, San Diego, USA). High-throughput sequencing was performed on an Illumina HiSeq platform (Illumina, San Diego, USA). Bioinformatics analysis including read trimming, filtering, and mapping to a reference genome was then conducted on the raw data by using HISAT2 tools. Further functional annotation and pathway analysis, utilizing resources like Kyoto Encyclopedia of Genes, Genomes (KEGG) and Gene Ontology (GO) and gene set enrichment analysis (GSEA), provide a comprehensive understanding of the biological significance of the identified genes. Differentially expressed genes were identified by setting a threshold at log2FC ≥ 1, FDR < 0.01 in TCG-treated BMMs, or log2FC ≥ 0.5, p-value < 0.05 in TCG-treated PMCs.

### Active Rap1 detection assay

To isolate active GTP-bound Rap1 (Rap1-GTP), we utilized the Rap1 Activation Assay Kit (Cell Biolabs, San Diego, USA) in accordance with the manufacturer's guidelines. Subsequently, SDS-PAGE analysis was performed on both pulldown and input samples to identify the presence of Rap1-GTP.

### Small molecule pull-down

The chemical synthesis process of biotin-linked TCG TCG-biotin) is shown in Additional file 2: Table S3. A pull-down experiment was performed coupled with mass spectrometry (MS) analysis to identify TCG-bound proteins. Briefly, lysates from BMMs were incubated with either biotin beads (Thermo Fisher, Waltham, USA) or TCG-biotin beads for 4 h at 4 °C. The proteins attached to the beads were subsequently isolated through SDS-PAGE. The gel's protein-containing band was meticulously excised, and in-gel digestion was performed. The resulting peptides underwent liquid chromatography-tandem mass spectrometry (LC–MS/MS) analysis for the identification of proteins.

### Virtual docking

Crystal structure of human Rap1b (PDB ID: 3X1W, chain A) was used for molecular docking study. The chain A of 3X1W was preprocessed by Protein Preparation Wizard. The ligand TCG was prepared by LigPrep and then docked into the protein by Induced Fit Docking. The resulting 3D structures of the ligand–protein complexes and the corresponding 2D ligand–protein interaction diagrams were visualized using Maestro.

### EdU assay

Cells were exposed to EdU for a 2-h duration at 37 °C. After fixed and permeabilized, a click reaction solution was applied for a 30-min staining period. Subsequently, cells were counterstained with Hoechst 33342 and observed using fluorescence microscopy to quantify EdU-positive cells and determine the percentage of proliferating cells.

### Flow cytometry

Flow cytometry was employed to detect PMCs apoptosis. PMCs were washed and centrifuged to achieve a single-cell suspension. This suspension was incubated with Annexin V to identify phosphatidylserine externalization. Propidium iodide, a nuclear dye, was added to distinguish apoptotic cells from necrotic cells. The samples were analyzed using a Thermo Fisher flow cytometer (Waltham, USA), and the fluorescence signals intensity and distribution were measured to quantitatively assess the extent of apoptosis.

### Measurement of extracellular acidification rate (ECAR)

For Seahorse extracellular flux analysis, the measurement of the ECAR was carried out as follows: Cells were seeded onto Seahorse assay plates and allowed to adhere. Then, the assay plates were loaded into the Seahorse XF Analyzer (Seahorse Biosciences, North Billerica, USA). During the experiment, a sequence of reagents was sequentially loaded including 10 mM glucose, 2 μM oligomycin, and 0.1 M 2-DG. The Seahorse XF Analyzer continuously monitors the changes in ECAR in real-time, allowing for the dynamic observation of cellular responses to metabolic perturbations.

### Lactate measurements

Lactate levels were quantified following the manufacturer's guidelines. Cells were seeded in 96-well plates and exposed to TCG for 24 h. Lactate levels in the culture medium were determined using the L-lactate assay kit (Eton Biosciences, Union, USA). The measurements were normalized to the total protein content in each well.

### Statistical analysis

The results were presented as mean ± SD. Statistical evaluations included independent t-tests and one-way ANOVA for datasets conforming to a normal distribution. A significance threshold of p < 0.05 was deemed statistically significant. Statistical analyses were conducted using SPSS 20.0 (SPSS, Inc., Chicago, USA) and GraphPad Prism 9.0 (GraphPad Software Inc., La Jolla, USA).

## Results

### TCG preserved articular cartilage and subchondral bone loss in DMM rats

**Fig. 1:**
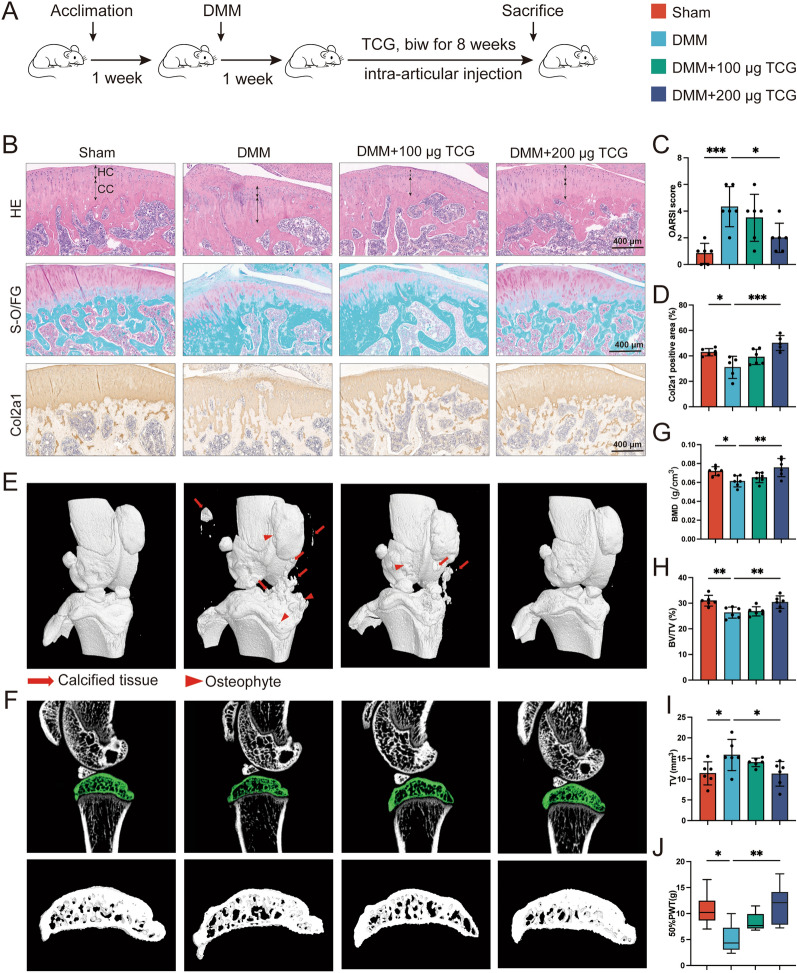
TCG preserved articular cartilage and subchondral bone loss in DMM rats. **A** Experimental flow chart. SD rats underwent DMM surgery to induce OA and were subsequently administered TCG injections for 8 weeks. **B** The knee joints of rats in each treatment groups were subjected to HE staining (first row), S–O/FG staining (second row) and Col2a1 immunostaining (third row). Hyaline cartilage (HC) and calcified cartilage (CC) were denoted by double-headed arrows. Scale bar = 400 µm. **C** The OA lesion was assessed following the OARSI grading system. **D** Positive staining area for Col2a1 was quantified. **E** 3D micro-CT images depicted pathological structural changes in the knees from each treatment group. Osteophytes and calcified tissues were highlighted in red arrows. **F** Sagittal views of medial compartment subchondral bone. **G**–**I** micro-CT analysis of tibial subchondral bone, including BMD, BV/TV, and TV. (H) Paw withdrawal threshold at 8 weeks after TCG treatment. Data are presented as mean ± SD (n = 6). *P < 0.05, **P < 0.01, ***P < 0.01

Chemical information of TCG is shown in Additional file 2: Table S4. To investigate the effect of TCG on articular cartilage loss in rat OA model, we conducted DMM surgery in male SD rats, followed by intra-articular injection of indicated doses of TCG (Fig. 1A). As observed in HE staining, sham-operated rats exhibited a unique cartilage structure composed of a Hyaline cartilage layer, calcified cartilage layer, and well-defined tidemark (Fig. 1B). In contrast, rats undergoing DMM surgery displayed compromised cartilage layer structure, characterized by indistinct boundaries between the transparent and calcified cartilage layers, thinning of the transparent cartilage layer, and thickening of the calcified cartilage layer. Additionally, S–O/F-G staining revealed a significant decrease in proteoglycan content (Fig. 1B). Notably, treatment with 200 µg TCG was observed to effectively restore the normal structure of the cartilage layer in DMM rats and also prevent the loss of proteoglycan (Fig. 1B). OARSI scores also indicated a marked improvement compared to vehicle-treated DMM controls (Fig. 1C). Consistently, immunohistochemical staining suggested that compared with DMM-operated rats, 200 µg TCG increased Col2a1 expression in articular cartilage layer (Fig. 1B, D). However, 100 µg TCG treatment did not yield similar effect as the 200 µg TCG.

Micro-CT analysis was further performed to evaluate the subchondral bone quality of DMM rats in different treatment groups. The 3D reconstruction revealed that DMM-operated rats displayed an increase in the formation of osteophytes and calcified tissue around the knee joint at 9 weeks following the surgery, while administration of 200 µg TCG decreased the formation of these tissues (Fig. 1E). Furthermore, TCG significantly elevated the tibial subchondral bone mineral density (BMD) and bone volume fraction (BV/TV), while decreasing tissue volume (TV) compared with DMM-operated rats (Fig. 1F–I). However, the effects were not as apparent in 100 µg TCG treated group. Von Frey hair test was then applied to evaluate the pain behavior, since the evidentiary correlation between subchondral bone remodeling and OA pain phenotypes [21]. As a result, 200 µg TCG treatment alleviated pain hypersensitivity caused by DMM (Fig. 1J). Therefore, our findings suggested that TCG treatment may protect DMM-induced articular cartilage by preserving subchondral bone remodeling, while alleviating the pain phenotype associated with OA.

### TCG suppressed RANKL and M-CSF induced osteoclast differentiation in vitro

**Fig. 2:**
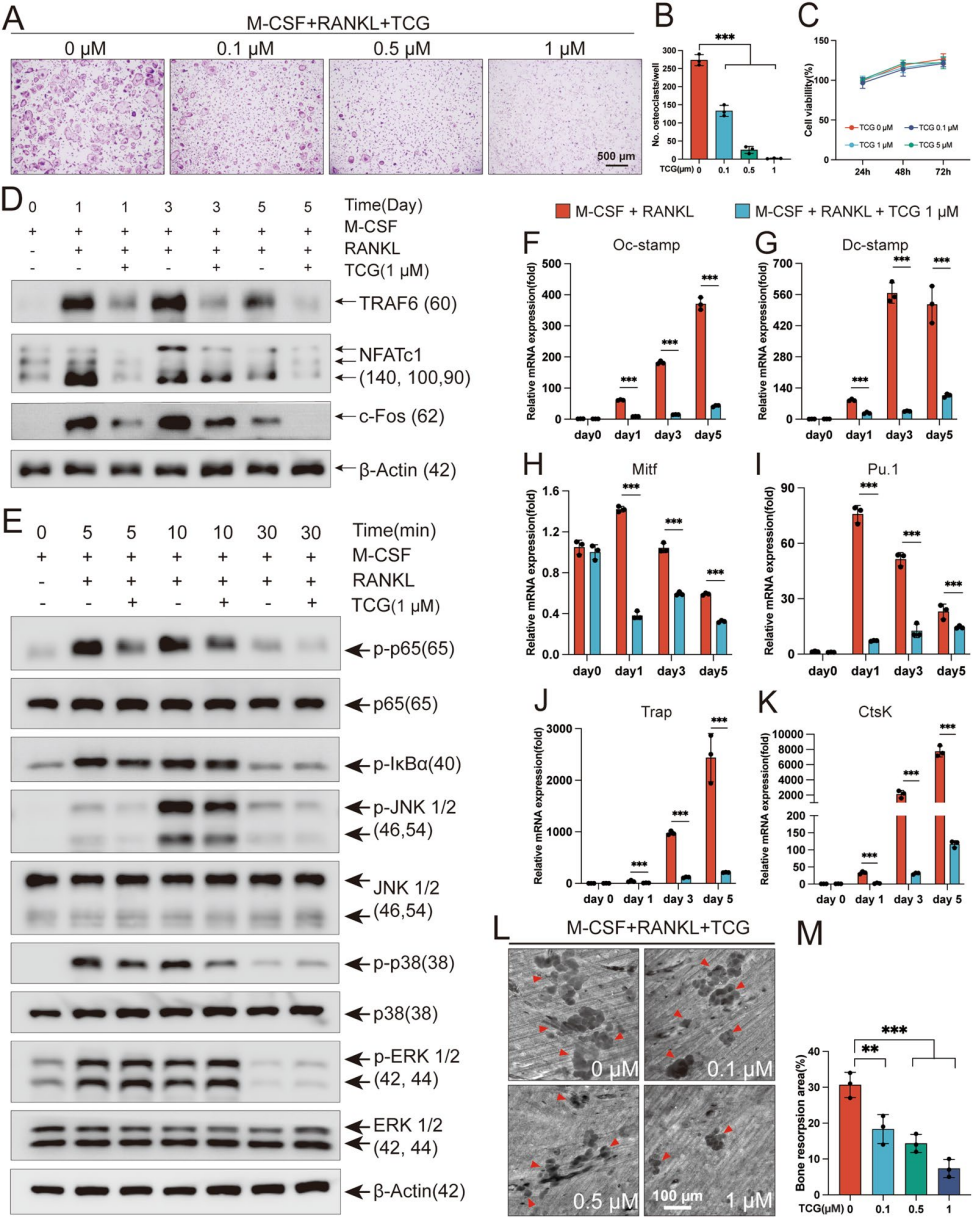
TCG suppressed RANKL and M-CSF induced osteoclast differentiation in vitro. **A**, **B** BMMs were induced into osteoclasts with RANKL and M-CSF while simultaneously treated with indicated doses of TCG. **A** Osteoclasts were visualized using TRAP staining (Scale bar = 500 µm). **B** Cells with more than 3 nuclei were considered as osteoclasts and included in statistics. **C** BMMs were treated with indicated doses of TCG for a specified period of time, the cytotoxicity of TCG on BMMs was assessed using CCK8 assay. **D**–**K** BMMs were induced with M-CSF and RANKL, and simultaneously treated with or without 1 µM TCG for a specified period of time. **D**, **E** Western blot measured the protein expression of TRAF6, NFATc1, c-Fos and the phosphorylation of MAPK and NFκB signaling molecules, the relative protein expression statistics were presented in Additional file 1: Fig. S1; **F**–**K** RT-PCR was conducted to assess the transcriptional levels of key genes involved in osteoclast differentiation. **L**, **M** Osteoclasts were seeded onto cortical bone slices and treated with indicated dose of TCG for 48 h. **L** Bone resorption pits appear cloud-like under confocal microscopy, as indicated by red arrows (Scale bar = 100 µm). **M** The area of bone resorption pits was quantified using ImageJ, and the percentage of bone resorption area was calculated by dividing the pit area by the total bone slice area. Data were presented as mean ± SD (n = 3). *P < 0.05, **P < 0.01, ***P < 0.01

Consider that the abnormal activation of osteoclasts is the primary cause of bone loss in subchondral bone, we further investigated the effect of TCG in osteoclastogenesis to illustrate the underling mechanism. Mouse primary BMMs was induced into osteoclasts in vitro with RANKL and M-CSF. As expected, TCG exhibited a dose-dependent suppression of osteoclastogenesis, wherein 1 µM TCG nearly abrogated the formation of osteoclasts (Fig. 2A, B). However, CCK-8 experiments confirmed that TCG had no detrimental effect on the proliferation of BMMs (Fig. 2C). Then, we detected the key intermediate signaling molecules involved in RANKL-induced osteoclastogenesis at 1, 3 and 5 days of RANKL stimulation. Our results indicated that 1 µM TCG significantly suppressed the amplification of TRAF6, NFATc1 and c-Fos (Fig. 2D, Additional file 1: Fig. S1A–C). Additionally, RANKL-mediated amplification of TRAF6 induce NFATc1/c-Fos through NF-κB and MAPK signals. It further indicated that 1 µM TCG inhibited the transient phosphorylation of p38 and JNK in the MAPK signaling pathway and phosphorylation of p65 and I-κBα in the NF-κB signaling pathway (Fig. 2E and Additional file 1: Fig. S1D–H). Besides, treatment with 1 μM TCG resulted in the suppression of nuclear translocation of p65 induced by RANKL (Additional file 1: Fig. S2). Moreover, we measured the effects of TCG in NFATc1/c-Fos induced early differentiation marker genes (Mitf, Pu.1), late differentiation marker genes (Oc-stamp and Dc-stamp) and bone resorption-marker genes (Trap and Ctsk) by qRT-PCR. As a result, 1 µM TCG inhibited the expressions of above-mentioned genes at 1, 3 and 5 days of RANKL stimulation (Fig. 2F–K). Consequently, bone resorption assay on bone slices also indicated that bone pits area was decreased due to indicated doses of TCG treatment (Fig. 2L, M). In conclusion, our results suggested that TCG inhibited RANKL signaling pathway related signaling molecules, and subsequently suppressed osteoclast formation and function.

### TCG inhibited osteoclastogenesis through suppressing Rap1 signaling pathway

**Fig. 3:**
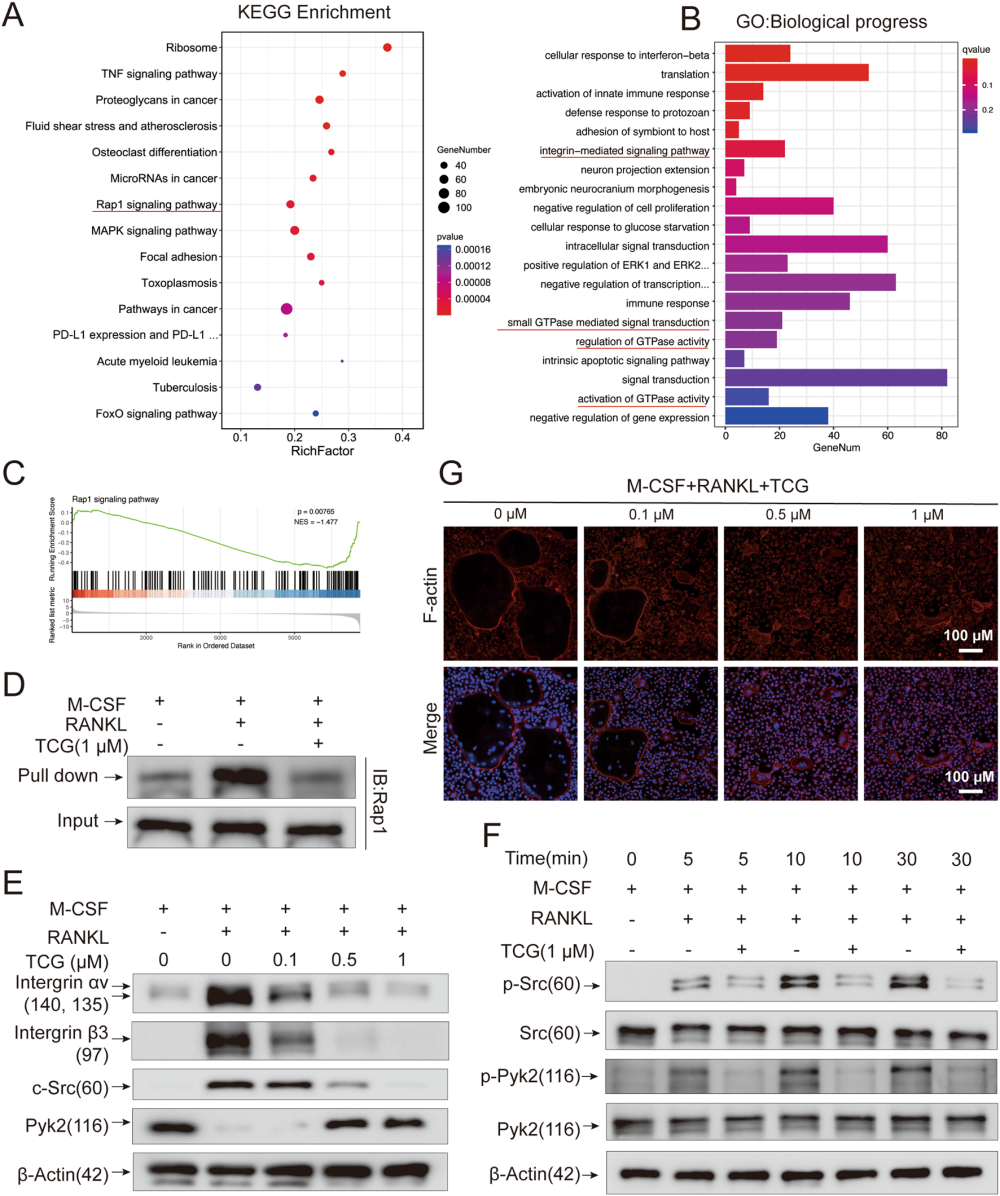
TCG inhibited osteoclastogenesis through suppressing Rap1 signaling pathway. **A**, **B** KEGG and GO analysis of differentially expressed genes between replicative BMMS treated with vehicle or 1 µM TCG for 3 days under RANKL and M-CSF stimulation. **C** GSEA analysis of Rap1 signaling pathway-related genes. **D** BMMs were stimulated with RANKL and M-CSF and simultaneously treated with or without 1 µM TCG for 15 min. Active Rap1 was detected using an active Rap1 detection kit. **E** BMMs were stimulated with RANKL and M-CSF and indicated doses of TCG for 3 days. Western blot measured the protein expression of integrin αv, integrin β3, c-Src and Pyk2. **F** BMMs were stimulated with RANKL and M-CSF and simultaneously treated with or without 1 µM TCG for an indicated period of time. Western blot assessed phosphorylated c-Src and Pyk2 protein expression. The relative protein expression statistics were presented in Additional file 1: Fig. S4A–F. **G** BMMs were induced to osteoclasts while simultaneously treated with indicated doses of TCG. Phalloidin staining showed the effect of indicated doses of TCG on F-actin ring formation (Scale bar = 100 µm)

To further investigate the mechanism by which TCG inhibited osteoclastogenesis, TCG-treated BMMs were subjected to RNA-seq analysis. The results showed apparent differences in the gene expression profiles between TCG-treated BMMs and the control group (Additional file 1: Fig. S3A). KEGG enrichment analysis of differential expressed genes indicated that they were primarily enriched in signaling pathways such as Rap1, MAPK and TNF (Fig. 3A). Besides, GO enrichment analysis suggested that they were primarily enriched in biological processes (Fig. 3B) related to small GTPases activity, small GTPase mediated signal transduction, integrins mediated signal pathway, and molecular functions (Additional file 1: Fig. S3B) such as GTP binding, GTPase activity. Coincidentally, Rap1 is a small Ras-like GTPase recognized for its role in regulating integrin-mediated cell adhesion and cytoskeleton formation in osteoclasts [22, 23]. A total of 51 differentially expressed genes associated with the Rap1 signaling pathway were identified, with the majority exhibiting down-regulation following TCG treatment (Additional file 1: Fig. S3C). These included Integrin Subunit Beta 3 (Itgb3) and Src, which are important intermediate signaling molecules in the Rap1 signaling pathway. Consistently, GESA analysis indicated that TCG treatment led to downregulation trend in Rap1 signaling pathway (Fig. 3C). Thus, we proposed that TCG might inhibit osteoclastogenesis by interfering with the Rap1 signaling pathway. To test this hypothesis, we used an activated Rap1 detection assay to investigate the impact of TCG on the RANKL induced conversion of Rap1-GDP to Rap1-GTP. As expected, RANKL stimulation led to the formation of Rap1-GTP, which was inhibited by TCG treatment (Fig. 3D).

Rap1-GTP activates integrin αvβ3, which regulates the formation of the osteoclast cytoskeleton. Thus, we applied western blot assay to detect changes in downstream signaling molecules. The results indicated that TCG dose-dependently suppressed the protein expression of integrin αv and integrin β3, following by the limited protein amplification of c-Src and degradation of Pyk2 upon RANKL stimulation (Fig. 3E, Additional file 1: Fig. S4A–D). Besides, TCG was observed to suppress the phosphorylation of c-Src and Pyk2 stimulated by RANKL in BMMs (Fig. 3F, Additional file 1: Fig. S4E, F), since Pyk2 and c-Src facilitate the integrin related cytoskeleton formation. To visualize the effects of TCG on osteoclast cytoskeleton, we conducted phalloidin staining to assess F-actin ring formation. Osteoclasts was observed to formed a complete F-actin ring structure with ruffled border. However, despite a small amount of cell fusion, normal F-actin ring formation was not achieved after 0.5 µm and 1 µM TCG treatment, as evidenced by changes in the size and shape of multinucleated cells (Fig. 3G). In short, we proposed that TCG primarily exerts its inhibition on osteoclast cytoskeletal formation by suppressing the activation of the Rap1 signaling pathway.

### TCG directly bound Rap1 in BMMs

**Fig. 4:**
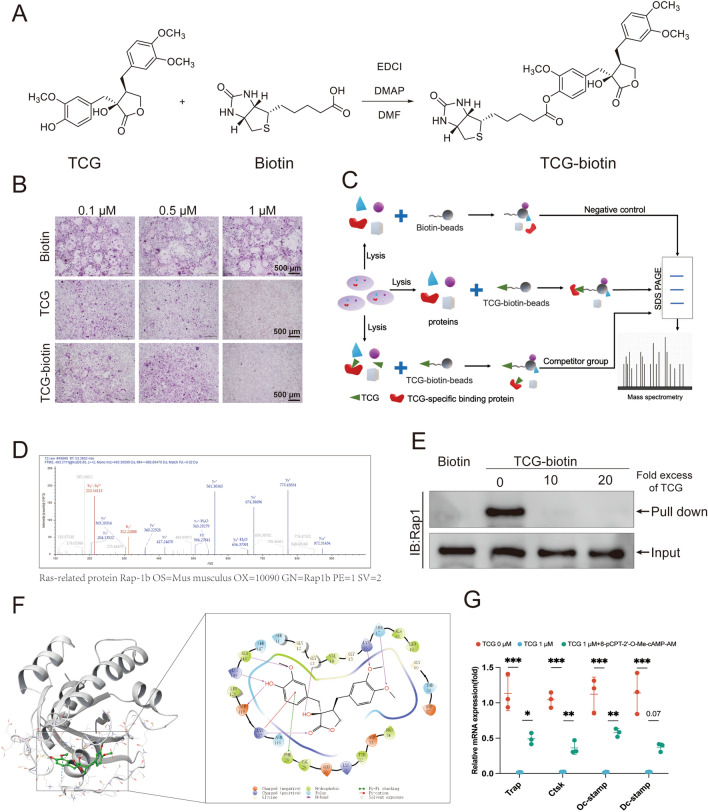
TCG directly bound Rap1 in BMMs. **A** Depiction of the chemical structure and chemical synthesis process of TCG-biotin. **B** BMMs were induced to osteoclasts while simultaneously treated with indicated doses of biotin, TCG or TCG-biotin separately. Osteoclasts were visualized using TRAP staining (Scale bar = 500 µm). **C** Schematic of the small molecule pull-down assay. Cell lysates of BMMs, with or without pre-treatment of TCG, were subjected to pull-down assay using biotin beads or TCG-biotin beads, followed by mass spectrometry analysis. **D** Representative mass spectrum of RAP1b. **E** Validation of the binding between TCG-biotin and RAP1 through small molecule pull-down assay and western blot. **F** Binding model of TCG-Rap1b. In panorama, TCG's atoms appeared as balls and rods, with carbon atoms in green, hydrogen atoms in white, and oxygen atoms in red. The entire protein was shown as a cartoon, while the amino acids at the binding site were represented as lines. In the enlarged view, ionic bonds are highlighted, with purple lines denoting hydrogen bonds, green lines indicating π–π stacking, and red lines representing π-cation interactions. **G** BMMs were stimulated with RANKL and M-CSF, and simultaneously co-treated with 1 µM 8-pCPT-2′-O-Me-cAMP-AM and 1 µM TCG for 48 h, transcript levels of genes involved in osteoclast differentiation were assessed by RT-PCR. Data were presented as mean ± SD (n = 3). *P < 0.05, **P < 0.01, ***P < 0.01

Up until now, our findings have revealed that TCG suppresses the activation of the Rap1 signaling pathway. This discovery has sparked our interest in elucidating its precise and direct molecular targets. To address this, we initiated a chemical synthesis process to attach a biotin moiety to TCG (Fig. 4A). The TRAP staining assay confirmed that TCG-biotin retained its inhibitory effect on osteoclast differentiation (Fig. 4B). Subsequently, a small molecule pull-down assay and the mass spectrometry analysis were performed. The sketch of technical route was illustrated in Fig. 4C. The results revealed a potential interaction between TCG-biotin and Rap1b (Fig. 4D, Additional file 3: Table S5). Western blot further confirmed a direct binding between TCG-biotin and Rap1, while biotin alone did not show such binding (Fig. 4E). Moreover, pre-blocking Rap1 with 10- or 20-fold excess of TCG prevented the binding of TCG-biotin to Rap1, which further demonstrated the specificity of the direct binding between TCG and Rap1b (Fig. 4E). Based on the strong affinity between TCG and Rap1b, it is speculated that there may be ionic bonds between them. Thus, we performed a molecular docking using the crystal structure of Rap1b. In the TCG-Rap1b complex, TCG formed 9 ionic bonds with 7 amino acids, including hydrogen bond with ALA148, LYS149, ASP119, LYS117, SER17, LYS16 and GLY13, π-π stacking with PHE28 and π-cation interaction with LYS117 (Fig. 4F).

Finally, we examined whether the Rap1 signaling pathway plays a role in TCG’s inhibition of osteoclast formation. 8-pCPT-2'-O-Me-cAMP-AM activates Rap1 GTPase intracellularly, thereby modulating biological effects associated with the Rap1 signaling pathway. qRT-PCR results showed that BMMs treated with a combination of 1 µM 8-pCPT-2'-O-Me-cAMP-AM and 1 µM TCG partially restored the inhibitory effect of TCG on osteoclast marker genes (Fig. 4G). These findings collectively suggested that TCG inhibited osteoclast differentiation by targeting Rap1 and inhibiting its activation.

### TCG promoted chondrocyte survival in vitro

**Fig. 5:**
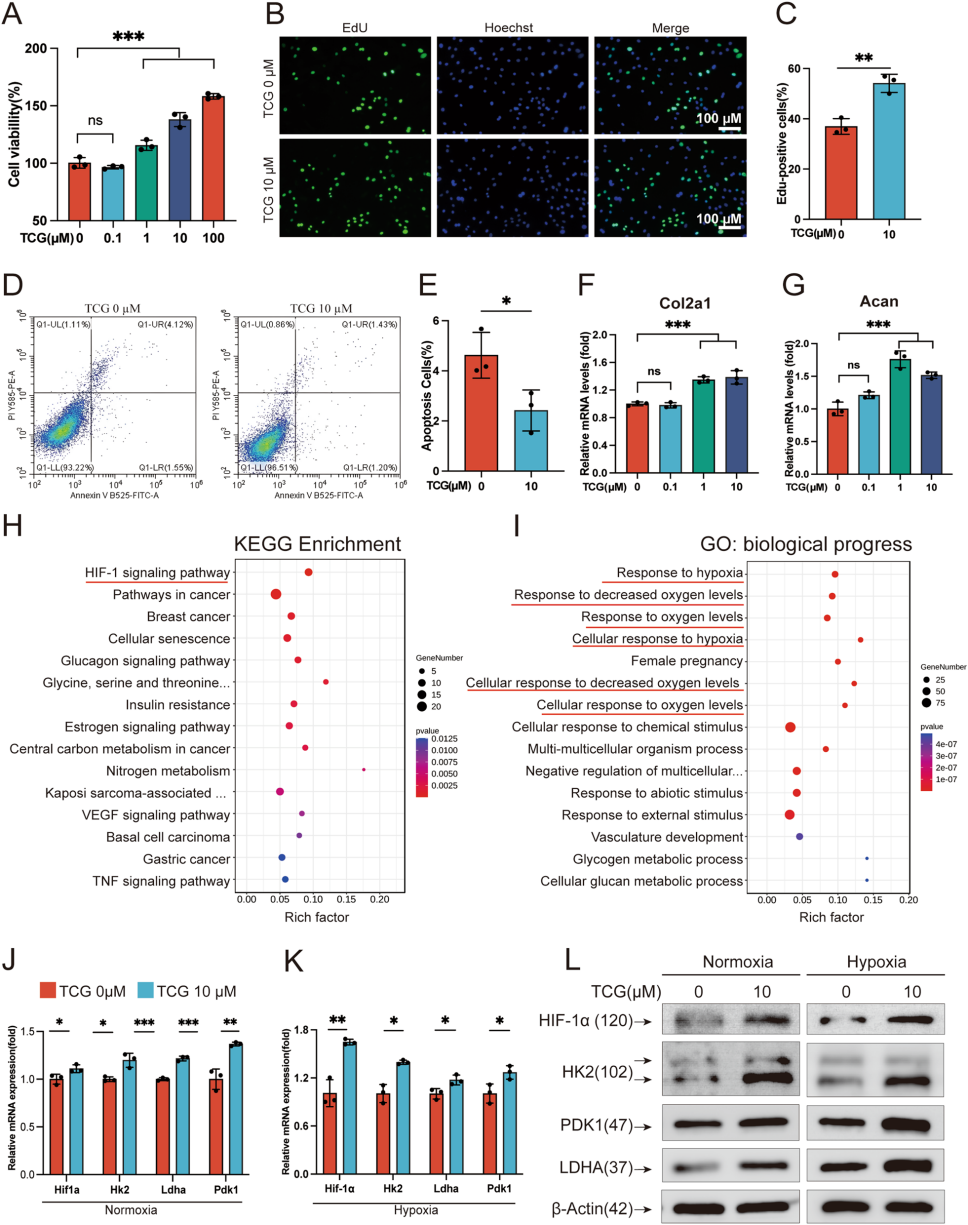
TCG promoted chondrocyte survival in vitro. **A**–**G** PMCs were treated with the indicated doses of TCG for 24 h. CCK8 assay (**A**) and EdU staining (**B**, **C**) was performed to assess the proliferation of PMCs (Scale bar = 100 µm); Flow cytometry (**D**, **E**) was used to detect the apoptotic rate of PMCs; RT-PCR (**F**, **G**) was employed to measure the transcriptional levels of ECM-related genes. **H**, **I** PMCs were treated with or without 10 µM TCG for 48 h, and RNA-sequence was conducted (n = 3 cells from 3 mice). Bubble chart showed the KEGG (**H**) and GO (**I**) analysis of differentially expressed genes. **J**, **L** PMCs were treated with 1 µM TCG under normoxic or hypoxic conditions for 24 h. RT-PCR was performed to assess the transcriptional levels of Hif1α and glycolysis-related genes under normoxic (**J**) and hypoxic (**K**) conditions, and western blot measured the protein expression of HIF1α and glycolytic enzymes (**L**). The relative protein expression statistics were presented in Additional file 1: Fig. S5A, B. Data were presented as mean ± SD (n = 3). *P < 0.05, **P < 0.01, ***P < 0.01

Chondrocytes are the sole cell type within cartilage and represent a crucial target for improving OA. To further illustrate the underline mechanism of TCG in treating OA, we wondered if TCG exert effects on cartilage chondrocytes. We isolated and cultured PMCs in vitro and treated with varying doses of TCG for 24 h. CCK8 assay was performed to assess cell viability. The results demonstrated a dose-dependent promotion of chondrocyte viability by TCG (Fig. 5A). To delve deeper into the impact of TCG on PMC proliferation, an EdU staining assay was conducted. The findings indicated a substantial rise in the percentage of EdU-positive cells upon exposure to 10 µM TCG, underscoring the proliferative effect of TCG on PMCs (Fig. 5B, C). Additionally, flow cytometry analysis demonstrated that 10 µM TCG significantly reduced PMCs apoptosis (Fig. 5D, E). Moreover, the influence of TCG on chondrocytes was corroborated through qRT-PCR analysis, revealing that the administration of 10 µM TCG led to an upregulation in the transcription of genes associated with extracellular matrix (ECM) production, including Col2a1 and Acan (Fig. 5F, G). Taken together, our data showed that TCG treatment enhanced PMCs survival and function by promoting PMCs proliferation and inhibiting apoptosis.

### TCG promoted chondrocyte survival through upregulating HIF1α and promoting glycolysis

**Fig. 6:**
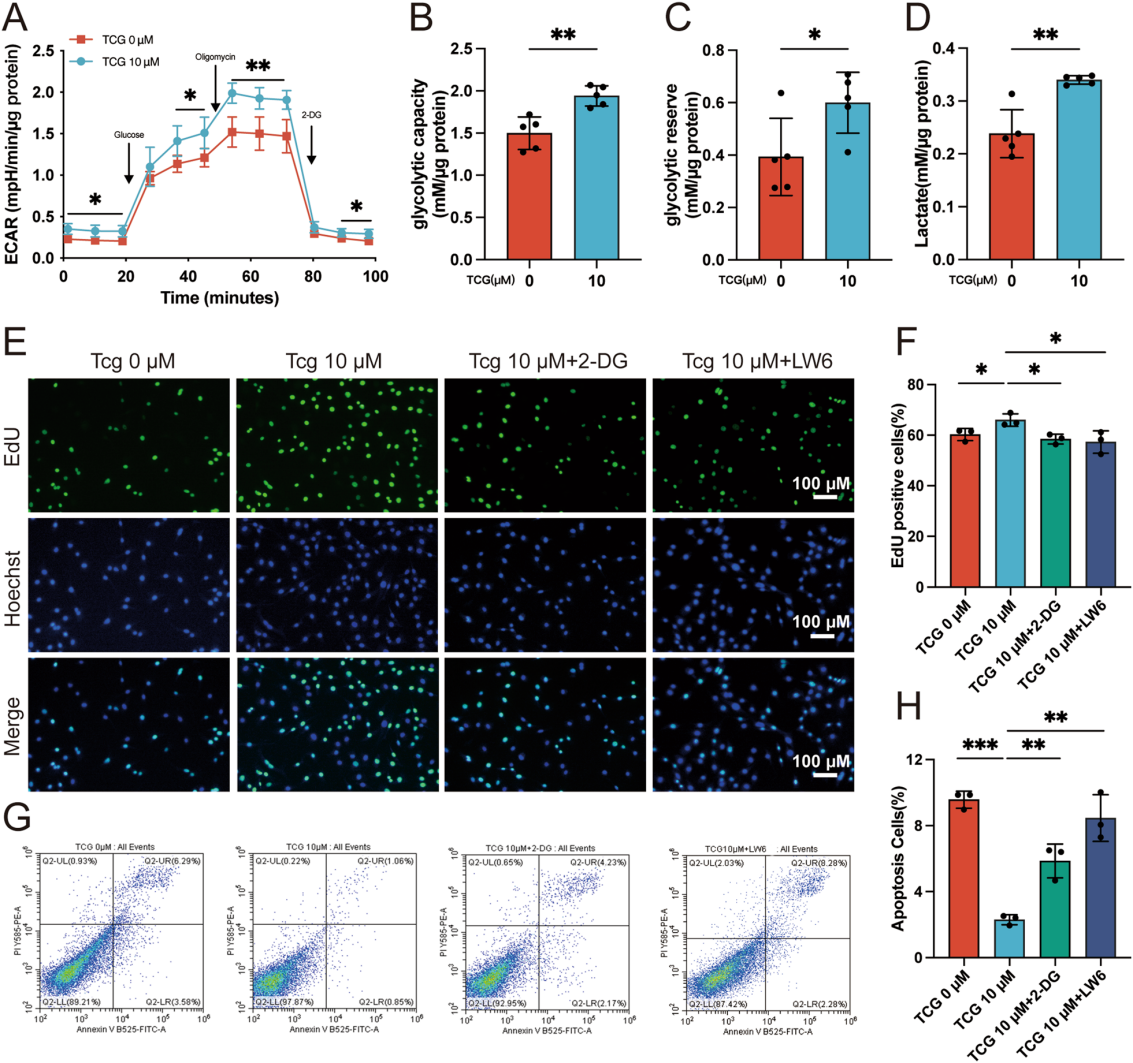
TCG promoted chondrocyte survival through upregulating HIF1α and promoting glycolysis. **A**–**D** PMCs were treated with or without 10 µM TCG for 24 h, and the ECAR (**A**) was measured using Seahorse assay to quantify glycolytic capacity (**B**) and glycolytic reserve (**C**); the lactate level in the cell culture medium was also measured (**D**). **E**–**H** PMCs were co-treated with 10 µM TCG and 16 mM 2-DG or 10 µM LW6 for 24 h under hypoxic. EdU staining was performed to assess cell proliferation rate (**E**–**F**) (Scale bar = 100 µm), and flow cytometry was conducted to detect cell apoptosis rate (**G**, **H**). Data are presented as mean ± SD (n = 3). *P < 0.05, **P < 0.01, ***P < 0.001

To elucidate the mechanism underlying TCG’s promotion of PMCs survival, we conducted RNA-seq on TCG-treated PMCs. KEGG and GO analysis were applied to highlight the pathways and biological processes in which TCG could affect PMCs. The KEGG showed that differentially expressed genes were mainly involved in HIF1, VEGF and TNF signaling pathways (Fig. 5H). The enriched biological progress terms mainly included cells response to hypoxia, response to oxygen level and so on (Fig. 5I). Coincidentally, articular chondrocytes are resident in a hypoxic environment. Low oxygen levels activate the HIF-1α signaling pathway, which drives chondrocytes to obtain energy through glycolysis under low oxygen conditions [24]. Then, qRT-PCR and western blot analyses were conducted to evaluate the transcription and protein expression of glycolysis-related enzymes in PMCs subjected to both normoxic and hypoxic conditions. In alignment with the RNA-seq findings, 10 µM TCG demonstrated a promotion of HIF1α and glycolytic enzymes (HK2, PDK1, LDHA) under both normoxic and hypoxic conditions (Fig. 5J–L). Additionally, we directly investigated the impact of TCG on the glycolytic capacity of PMCs through Seahorse real-time metabolic analysis. The results indicated that 10 µM TCG treatment enhanced the maximal glycolytic capacity and glycolytic reserve of PMCs (Fig. 6A–C). Lactate is a glycolytic end product. The content of lactate in the supernatant of PMCs culture medium were significantly increased by 10 µM TCG treatment for 24 h (Fig. 6D). To further verify the role of HIF1α and glycolysis in the regulation of PMCs by TCG, we treated PMCs by 10 µM TCG with/without HIF1α inhibitor LW6 (10 µM) or glycolysis inhibitor 2-DG (16 mM) under hypoxia. EdU staining and flow cytometry indicated that TCG promoted the proliferation of PMCs and inhibited their apoptosis under hypoxia. In contrast, LW6 and 2-DG reversed its effect (Fig. 6E–H). The above results suggested that TCG promotes the glycolysis of PMCs by upregulating HIF1α in both hypoxia and normoxia environments, thereby promoting PMCs proliferation and inhibiting apoptosis.

## Discussion

*T. jasminoides* has been traditionally used in Chinese Pharmacopeia for the treatment of OA, rheumatoid arthritis, backache, pharyngitis, bruises with confirmed clinical effects [25]. However, the precise mechanism of *T. jasminoides* in treating OA remains unknown. Our previous study using mass spectrometry and revealed that TCG is a major compound of *T. jasminoides* [14]. In this study, we further elucidated that TCG not only reduced subchondral bone loss by inhibiting osteoclasts differentiation and function, but also enhanced the survival of PMCs, thereby protecting articular cartilage during the progression of OA.

We first evaluated the anti-OA effects of TCG on a joint instability-induced osteoarthritis model (DMM model), which allows for investigation of structural and biological changes in the disease process and evaluation of the efficacy of OA drug candidates [26]. Our results indicated that treatment with 200 µg TCG effectively countered subchondral bone loss resulting from DMM surgery (Fig. 1F–I). Since subchondral bone microfractures heighten pain sensitivity through nerve fiber penetration [27]. The von Frey test also suggested TCG's capability to mitigate hyperalgesia in OA rats by preserving subchondral bone integrity (Fig. 1J). Besides, histological staining confirmed 200 µg TCG's concurrently protection on articular cartilage loss (Fig. 1B–D). Thus, the aforementioned outcomes in vivo suggested that TCG might inhibit abnormal subchondral bone remodeling, consequently preventing OA-associated subchondral bone loss and preserving articular cartilage. To further elucidate the underlying mechanism, we explored the impact of TCG on osteoclastogenesis. Given that abnormal joint stress in the early stages of OA can activate osteoclasts, contributing to excessive bone resorption and subsequent subchondral bone loss [28], investigating the effects on osteoclastogenesis becomes crucial. Our study demonstrated that TCG effectively inhibited osteoclast differentiation and bone resorption in vitro (Fig. 2A, B, L, M). Consistently, the transcription of osteoclastogenesis related genes and protein expression of signal molecules were also reduced (Fig. 2D–K).

Besides, RNA-seq results provided more important information on the underling mechanism by which TCG inhibited osteoclastogenesis. The enrichment analysis revealed that TCG may exert its inhibitory effects by modulating Rap1 and integrin signaling pathways (Fig. 3A–C). Rap1 signaling pathway is involved in various cellular processes. In osteoclastogenesis, the Rap1 signaling pathway is involved in integrin-mediated osteoclast skeleton formation [23, 29]. Therefore, we examined the Rap1 activation and subsequent integrin related downstream signaling pathways by western blot. Our findings demonstrated that TCG inhibited RANKL induced activation of Rap1 (Fig. 3D) and decreased the protein expression of integrin αv and integrin β3 (Fig. 3E). This observation aligned with the diminished phosphorylation of c-Src at Tyr416 and Pyk2 at Tyr402 (Fig. 3F). Phalloidin staining also confirmed that BMMs failed to form a normal cytoskeleton after TCG treatment (Fig. 3G). Integrins, particularly αvβ3, play a crucial role in osteoclastogenesis by promoting podosomes formation and facilitating cytoskeletal rearrangement [30]. In osteoclasts, active Rap1 triggers the inside-out activation and clustering of integrin αvβ3, leading to the recruitment and activation of c-Src and Pyk2 complex [31, 32]. c-Src and Pyk2 are both present and localized in podosomes, actively participating in osteoclast skeletal formation and various cellular functions such as fusion and migration. Thus, our study revealed that TCG exerts inhibitory effects on osteoclastogenesis and bone resorption through the Rap1/integrin αvβ3/c-Src/Pyk2 signaling cascade, providing a deeper understanding of the underlying mechanisms that underlie its anti-OA effects.

More importantly, our pull-down and MS experiments also revealed the direct binding between TCG and Rap1 (Fig. 4A–D). It was further validated by western blot and computer-based virtual docking (Fig. 4E, F). Moreover, as a Rap1 activator, 8-pCPT-2′-O-Me-cAMP-AM counteracted the inhibitory effect of TCG on osteoclasts, which indicated TCG exerted the anti-osteoclastogenesis effects by targeting Rap1 (Fig. 4G). Rap1 belongs to the Ras family of small GTPases. In a recent study by Zou et al., osteoclast-specific Rap1 deletion mice were generated, revealing that Rap1 knockout (KO) mice displayed osteopetrosis and compromised osteoclast function [23]. Small GTPases were demonstrated to play pivotal roles in the differentiation, function, and survival of osteoclasts [33]. For instance, osteoclast-specific small GTPase knockout mouse models, including Cdc42, Rac1, Rac2 and Rap1, have been observed to exhibit abnormal bone phenotype [34]. Thus, small GTPases have emerged as prominent targets for developing osteoclast inhibitors [35]. This underscores the demand for novel small GTPases-targeted osteoclast inhibitors that selectively target small GTPases. Our research demonstrated TCG's direct binding to Rap1 and its inhibitory effect on Rap1 activation and downstream signaling pathway, indicating its potential as a foundational compound for developing small GTPases-targeted osteoclast inhibitors. It also revealed that targeting Rap1 could be an option for the diseases related to bone resorption.

Chondrocytes are the only cell type in mature cartilage tissue and secrete ECM. It’s well known that enhancing chondrocyte survival is imperative to preserving ECM integrity and mitigating OA progression. Unfortunately, the limited replication capacity of chondrocytes limits the inherent healing ability of cartilage after injury. What's worse, the dysfunction and apoptosis of chondrocytes in OA progress can trigger active ECM loss, which in turn alters the biomechanical milieu around chondrocytes and further exacerbates the progression of the disease [36]. In the current research, TCG has been shown to promote chondrocyte proliferation and inhibit apoptosis in vitro under normoxia or hypoxia condition (Figs. 5A–C, 6E, F, 5D, E, 6G, H), providing insight into its mechanism on ECM production both in vivo (Fig. 5F, G) and in vitro (Fig. 1B–D). This further enhances our understanding of TCG's potential in alleviating OA, shedding light on its mechanism of action and its impact on ECM production.

To investigate how TCG modulates PMCs, we initially performed RNA-seq on TCG-treated PMCs. Our analysis revealed no significant alterations in genes related to Rap1 signaling pathway, including Rap1a, Rap1b, and downstream signaling molecules such as Tln1, Tln2 (Data not shown). Based on these findings, we postulate that TCG may not exert regulatory effects on PMCs through Rap1 signaling pathway. However, the RNA-seq analysis revealed a potential influence on the HIF1 signaling pathway, as supported by GO analysis indicating changes in processes linked to cellular responses to hypoxia (Fig. 5H, I). Articular cartilage naturally resides in a hypoxic environment due to its lack of blood vessels and limited oxygen diffusion from synovial fluid. The hypoxic environment triggers cells reactions and adaptive mechanisms to ensure chondrocyte survival including the elevation of hypoxia-inducible factors (HIFs) [37]. HIF1α initiates glycolytic metabolism to support the metabolic requirements of proliferation and matrix synthesis [38]. It has emerged as a crucial therapeutic target for OA by regulating chondrocyte metabolism. Our findings suggest that TCG treatment of PMCs results in an upregulation of HIF1α and glycolytic enzymes, including HK2, PDK1, and LDHA (Fig. 5J–L), which was verified by Seahorse experiments and the detection of glycolytic end product lactate (Fig. 6A–D). This was further validated by the coculture experiment with HIF1α inhibitor LW6 or glycolysis inhibitor 2-DG (Figure E–H), since LW6 or 2-DG effectively attenuated the beneficial effects of TCG on cartilage. Our results demonstrate that TCG enhances HIF1α signaling and augments glycolysis in chondrocytes under hypoxic conditions to promote cell proliferation, providing more insights into the protective effect of TCG on articular cartilage.

## Conclusion

**Fig. 7:**
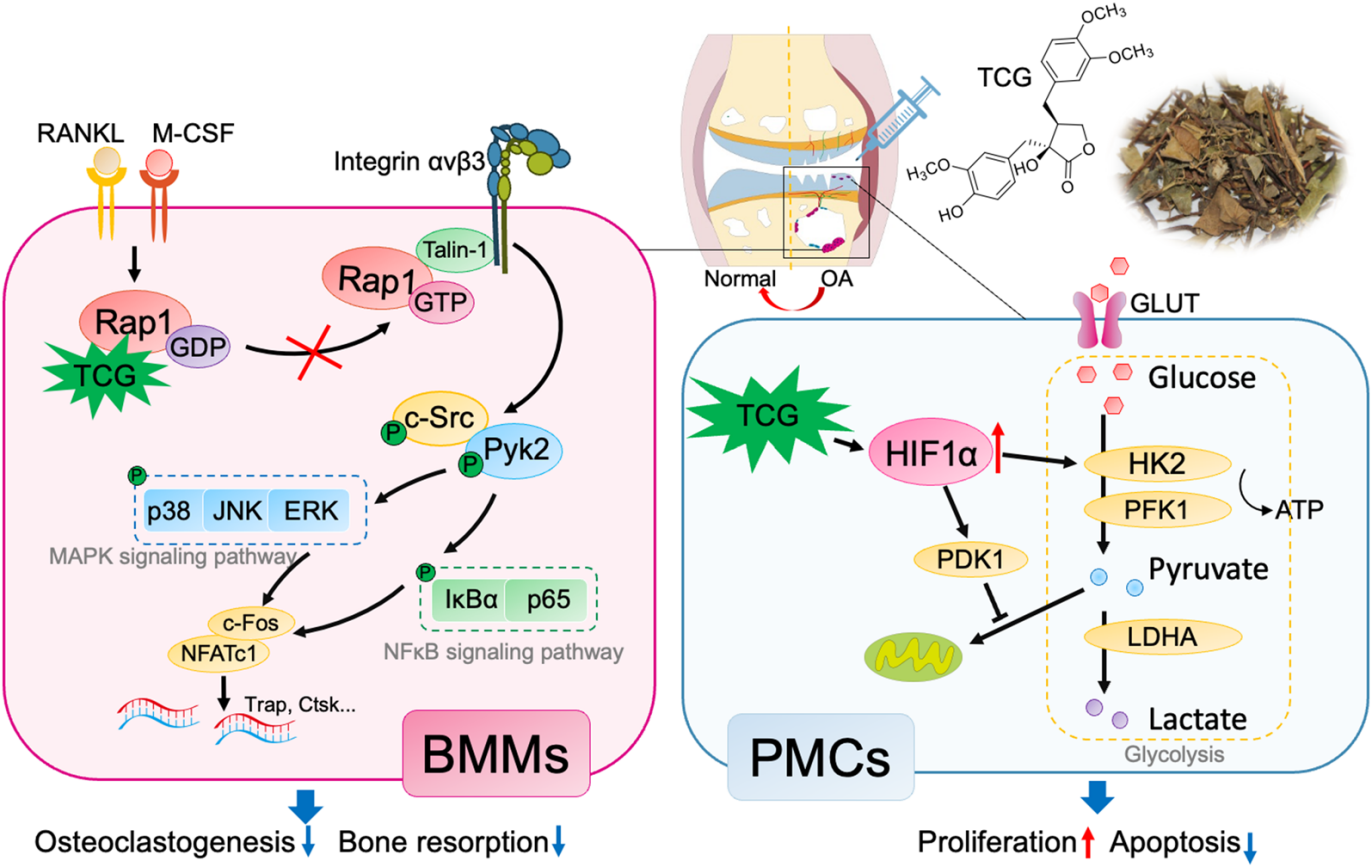
The schematic diagram of the mechanisms of TCG in BMMs and PMCs. TCG inhibited osteoclastogenesis and function through binding to Rap1 and inhibiting Rap1/integrin αvβ3/c-Src/Pyk2 signaling cascade in BMMs. Besides, TCG promoted the proliferation of PMCs while suppressing apoptosis by upregulating HIF1α, and thereby promoting glycolysis

This study presented TCG, a small molecule natural product, as a potential therapeutic agent for relieving OA. TCG exerted inhibitory effects on osteoclast differentiation by binding to Rap1 and inhibiting Rap1 activation, followed by thus preventing subchondral bone loss. Moreover, TCG enhanced chondrocyte survival by upregulating HIF1α and promoting glycolysis (Fig. 7). These dual mechanisms collectively provide a novel approach to prevented against cartilage degradation in a rat OA model. Our study not only highlights TCG's therapeutic promise in OA treatment, and advance our understanding of *T. jasminoides*, but also emphasizes Rap1 as a potential target for addressing bone resorption-related diseases.

### Supplementary Information

**Additional file 1: Figure S1.** The relative protein expression statistics of western blot in Fig. 2D and Fig. 2E. **Figure S2.** TCG inhibited RANKL-induced nuclear translocation of p65 in BMMs. **Figure S3.** The RNA-seq results of TCG treated BMMs. **Figure S4.** The relative protein expression statistics of western blot in Fig. 3E and Fig. 3F. **Figure S4.** The relative protein expression statistics of western blot in Fig. 5L.

**Additional file 2: Table S1.** Primers used in real-time PCR. **Table S2.** Primary antibodies used for western blot and immunofluorescence. **Table S3.** Chemical synthesis of TCG-biotin. **Table S4.** Chemical structure of TCG.

**Additional file 3: Table S5.** Protein identification based on LC–MS/MS analysis.

## Data Availability

The datasets used and/or analyzed during the current study are available from the corresponding author on reasonable request.

## Abbreviations

OA: Osteoarthritis
DMOADs: Disease-modifying OA drugs
*T. jasminoides*: *Trachelospermum jasminoides* (Lindl.) Lem
TCG: Trachelogenin
DMM: Destabilization of the medial meniscus
S–O/FG: Safranin O and fast green
KEGG: Kyoto encyclopedia of genes and genomes
GO: Gene ontology
GSEA: Gene set enrichment analysis
TRAP: Leukocyte acid phosphatase
LC–MS/MS: Liquid chromatography-tandem mass spectrometry
ECAR: Extracellular acidification rate
PWT: Paw withdrawal threshold
BMMs: Bone marrow-derived macrophages
M-CSF: Macrophage-stimulating factor
RANKL: Nuclear factor kappa-B ligand
PMCs: Primary mouse chondrocytes
Rap1: Ras association proximate 1
HIFs: Hypoxia-inducible factors
