# Supplementary material for: Trachelogenin alleviates osteoarthritis by inhibiting osteoclastogenesis and enhancing chondrocyte survival
Source: Chin Med. 2024 Mar 1;19:37. doi: 10.1186/s13020-024-00909-x (PMC10905921; doi:10.1186/s13020-024-00909-x)
Supplement: Supplementary file 1 — Additional file 1: Figure S1. The relative protein expression statistics of western blot in Fig. 2D and Fig. 2E. Figure S2. TCG inhibited RANKL-induced nuclear translocation of p65 in BMMs. Figure S3. The RNA-seq results of TCG treated BMMs. Figure S4. The relative protein expression statistics of western blot in Fig. 3E and Fig. 3F. Figure S4. The relative protein expression statistics of western blot in Fig. 5L. [file 13020_2024_909_MOESM1_ESM.docx]

**Additional Figure S1-S5**

**
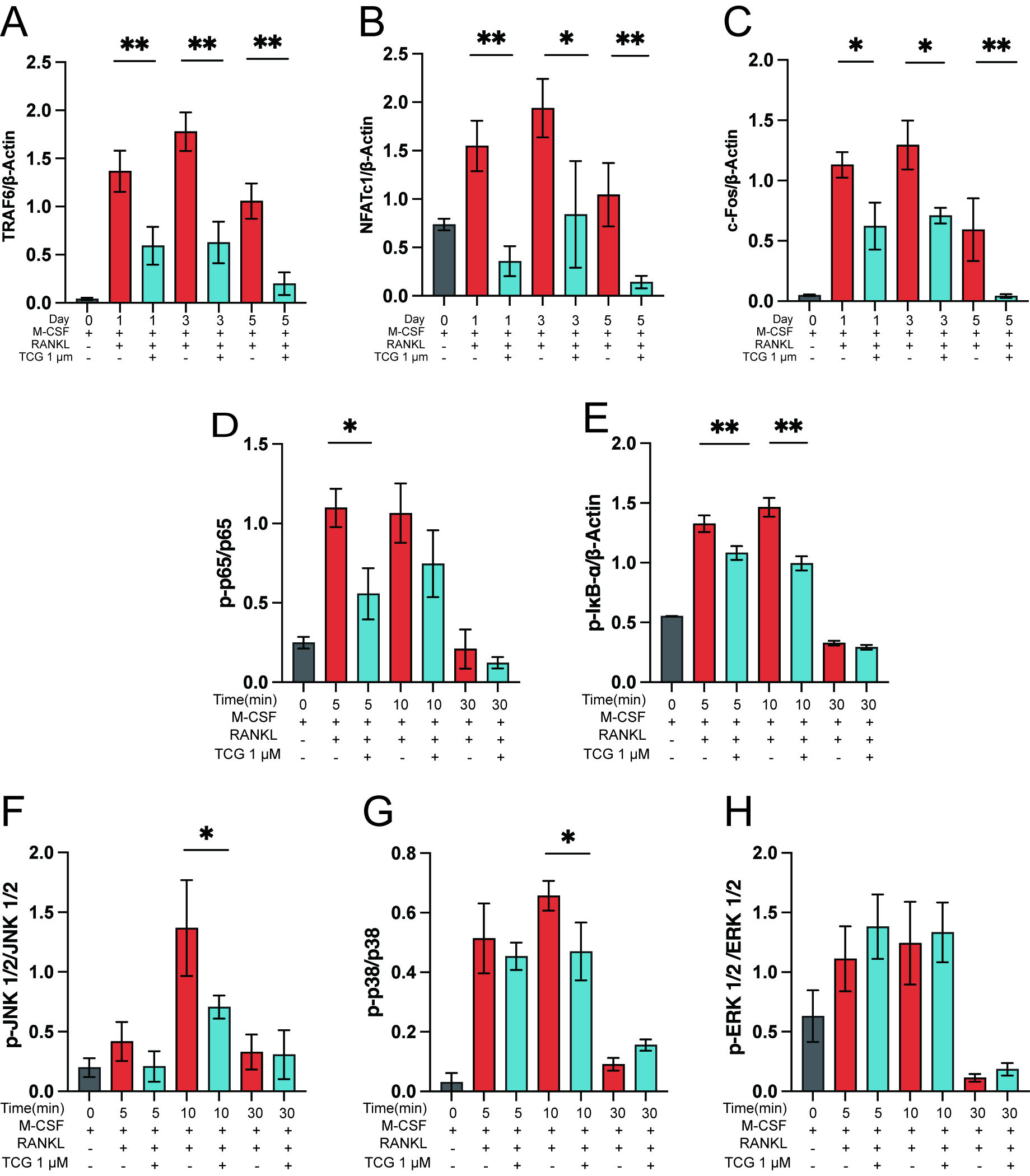
**

**Figure S1.**

(A-C) The statistical analysis of relative protein expression in western blots presented in Figure 2D was performed by normalizing the gray scale of TRAF6, NFATc1 and c-Fos to β-Actin. (D-H) The statistical analysis of relative protein expression in Western blots presented in Figure 2E was conducted by normalizing the gray scale of p-p65 to p65, and p-IκBα to β-Actin, p-JNK 1/2 to JNK 1/2, p-p38 to p38, p-ERK 1/2 to ERK1/2 individually. Significance levels are denoted as follows: * P<0.05, ** P<0.01, *** P<0.01 (n=3).

**
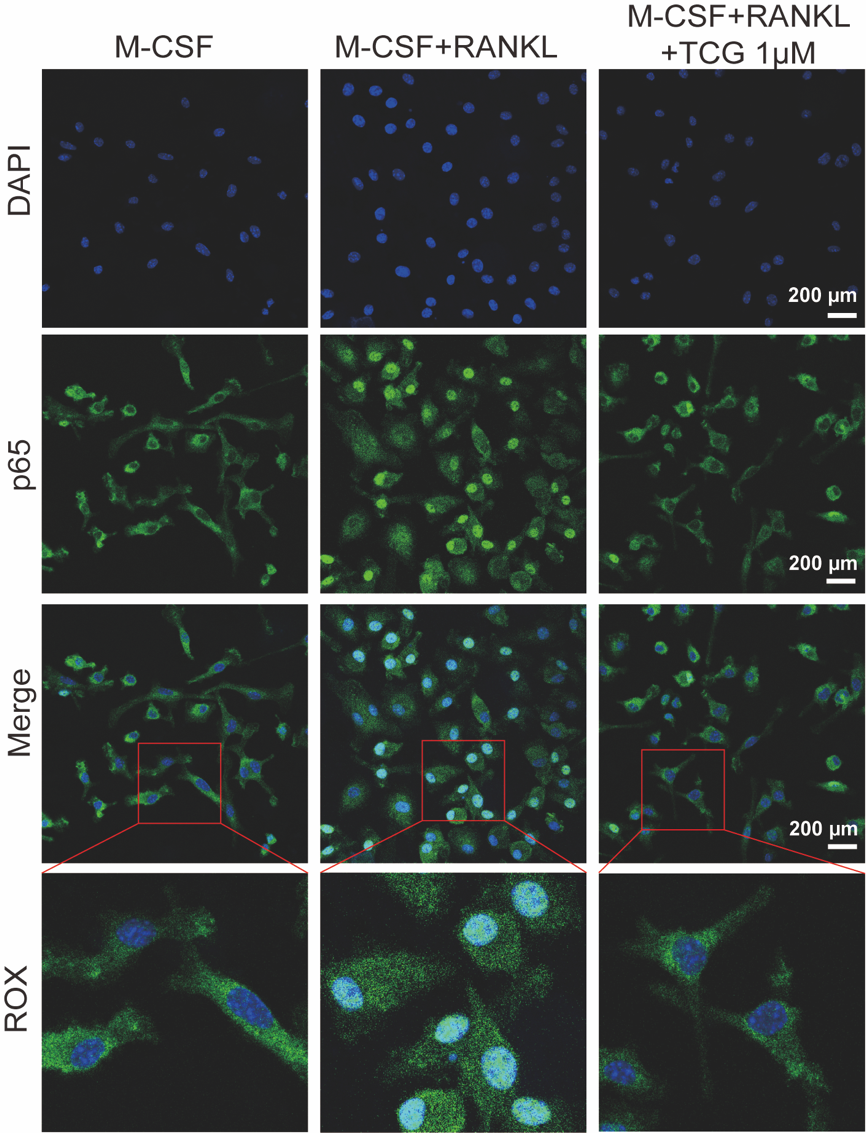
**

**Figure S2.**

TCG inhibited RANKL-induced nuclear translocation of p65 in BMMs. BMMs were stimulated with RANKL and M-CSF, and simultaneously treated with or without 1 µM TCG for 60 minutes. The cells were ﬁxed and stained for immunoﬂuorescence with p65 antibody (Scale bar = 200 µm).

**
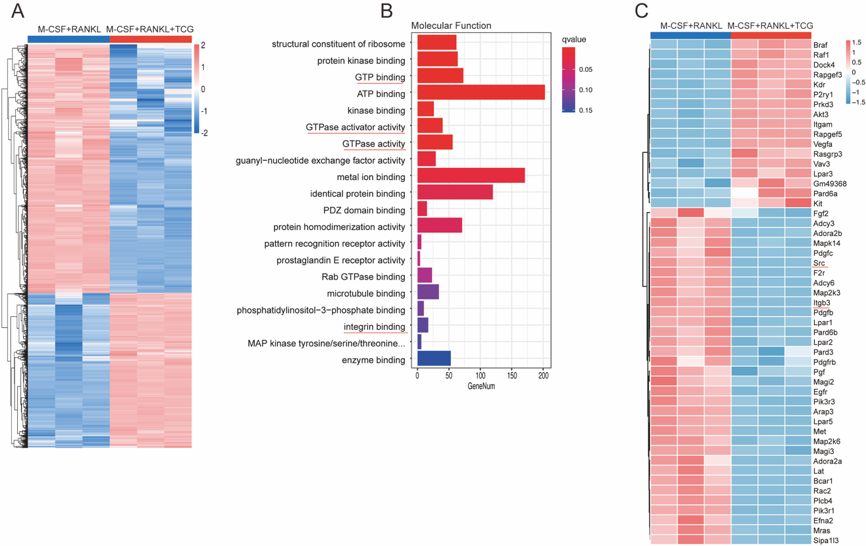
**

**Figure S3**

BMMs were stimulated with RANKL and M-CSF, and simultaneously treated with or without 1 µM TCG for 3 days, and RNA-sequence was conducted (n=3 cells from 3 mice). (A) Heatmap showed the differentially expressed transcripts between two groups. (B) GO (molecular function) analysis of differentially expressed genes. (C) Heat map depicted differentially expressed genes associated with the Rap1 signaling pathway.

**
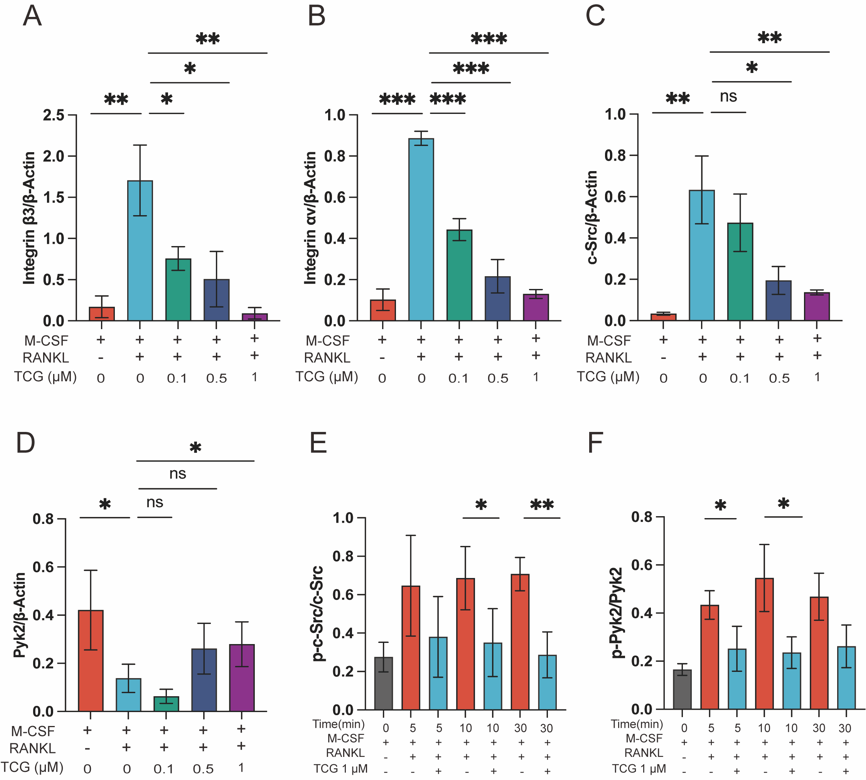
**

**Figure S4**

(A-D) The statistical analysis of relative protein expression in western blots presented in Figure 3E was performed by normalizing the gray scale of integrin β3, integrin αv, c-Src, Pyk2 to β-Actin. (E-F) The statistical analysis of relative protein expression in Western blots presented in Figure 3F was conducted by normalizing the gray scale of p-c-Src to c-Src, p-Pyk2 to Pyk2. Significance levels are denoted as follows: * P<0.05, ** P<0.01, *** P<0.01 (n=3).

**
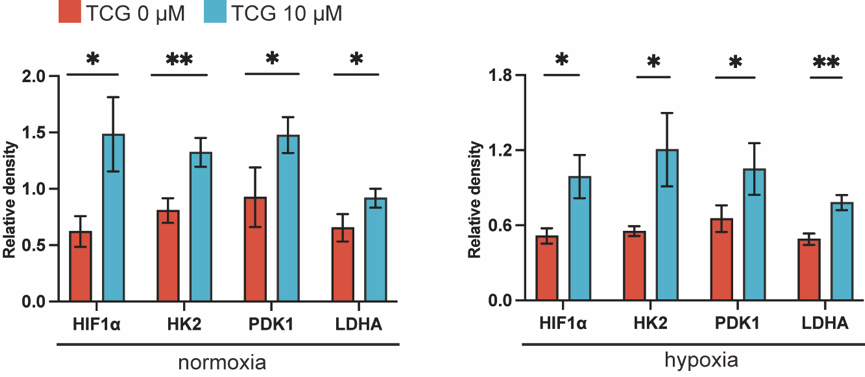
**

**Figure S5**

The statistical analysis of relative protein expression in western blots presented in Figure 5L was performed by normalizing the gray scale of HIF-1α, HK2, PDK1, LDHA to β-Actin. Significance levels are denoted as follows: * P<0.05, ** P<0.01, *** P<0.01 (n=3).
