# Supplementary material for: Trachelogenin alleviates osteoarthritis by inhibiting osteoclastogenesis and enhancing chondrocyte survival
Source: Chin Med. 2024 Mar 1;19:37. doi: 10.1186/s13020-024-00909-x (PMC10905921; doi:10.1186/s13020-024-00909-x)
Supplement: Supplementary file 2 — Additional file 2: Table S1. Primers used in real-time PCR. Table S2. Primary antibodies used for western blot and immunofluorescence. Table S3. Chemical synthesis of TCG-biotin. Table S4. Chemical structure of TCG. [file 13020_2024_909_MOESM2_ESM.docx]

**Table S1. Primers used in real-time PCR**

| Gene | Primer sequences | |
| --- | --- | --- |
| Trap | Forward | 5'-CACTCCCACCCTGAGATTTGT-3' |
|  | Reverse | 5'-CATCGTCTGCACGGTTCTG-3' |
| Ctsk | Forward | 5'-GAAGAAGACTCACCAGAAGCAG-3' |
|  | Reverse | 5'-TCCAGGTTATGGGCAGAGATT-3' |
| Dc-stamp | Forward | 5'-GGGGACTTATGTGTTTCCACG-3' |
|  | Reverse | 5'-ACAAAGCAACAGACTCCCAAAT-3' |
| Oc-stamp | Forward | 5'-CTGTAACGAACTACTGACCCAGC-3' |
|  | Reverse | 5'-CCCAGGCTTAGGAAGACGAAG-3' |
| Pu.1 | Forward | 5'-AGGAGTCTTCTACGACCTGGA-3' |
|  | Reverse | 5'-GAAGGCTTCATAGGGAGCGAT-3 |
| Mitf | Forward | 5'-CAAATGGCAAATACGTTACCCG-3' |
|  | Reverse | 5'-CTCCCTTTTTATGTTGGGAAGGT-3' |
| Acan | Forward | 5'-CCTGCTACTTCATCGACCCC-3' |
|  | Reverse | 5'-AGATGCTGTTGACTCGAACCT-3' |
| Col2a1 | Forward | 5'-TGAAGACCCAGACTGCCTCAA-3' |
|  | Reverse | 5'-AGCCGCGAAGTTCTTTTCTCC-3' |
| Comp | Forward | 5'-CTGCAGGACTGGCTCAAAGG-3' |
|  | Reverse | 5'-CATGGTCAGGATGTATGGTGGT-3' |
| Hk2 | forward | 5'-CAGGGTCTGAGCAAGGAGAC-3' |
|  | reverse | 5'-AAGATCCAAGGCCAGGAAGT-3' |
| Pdk1 | forward | 5'-GGACTTCGGGTCAGTGAATGC-3' |
|  | reverse | 5'-TCCTGAGAAGATTGTCGGGGA-3' |
| Hif1α | forward | 5'-AGCACAGTTACAGTATTCCAGCAGAC-3' |
|  | reverse | 5'-TCATCAGTGGTGGCAGTGGTAGT-3' |
| β-actin | forward | GCTGTGCTATGTTGCTCTAGACT |
|  | reverse | GTTGGCATAGAGGTCTTTACGGA |

**Table S2. Primary antibodies used for western blot and immunofluorescence**

| Primary Antibody | Company | Catalog No. | Host species |
| --- | --- | --- | --- |
| c-Fos | Cell Signaling Technology | #2250 | Rabbit |
| NFATc1 | Santa Cruz | sc-7294 | Rabbit |
| TRAF6 | Santa Cruz | sc-8409 | Rabbit |
| HSP90 | Cell Signaling Technology | #4874 | Mouse |
| p65 | Cell Signaling Technology | #3034 | Rabbit |
| Phospho-p65 | Cell Signaling Technology | #3033 | Rabbit |
| Phospho-iκBα | Cell Signaling Technology | #5209 | Rabbit |
| JNK1/2 | Cell Signaling Technology | #9252 | Rabbit |
| Phospho- JNK1/2 | Cell Signaling Technology | #4668 | Rabbit |
| p38 | Cell Signaling Technology | #8690 | Rabbit |
| Phospho-p38 | Cell Signaling Technology | #4511 | Rabbit |
| ERK1/2 | Cell Signaling Technology | #4695 | Rabbit |
| Phospho-ERK1/2 | Cell Signaling Technology | #4370 | Rabbit |
| β-actin | Cell Signaling Technology | #3700 | Rabbit |
| Integrin αV | Cell Signaling Technology | #4711 | Rabbit |
| Integrin β3 | Cell Signaling Technology | #29462 | Rabbit |
| Src | Cell Signaling Technology | #2109 | Rabbit |
| Phospho-Src (Tyr416) | Cell Signaling Technology | #59548 | Rabbit |
| Pyk2 | Cell Signaling Technology | #3480 | Rabbit |
| Phospho-Pyk2 (Tyr402) | Cell Signaling Technology | #3291 | Rabbit |
| Hk2 | Cell Signaling Technology | #2867 | Rabbit |
| Ldha | Cell Signaling Technology | #2012 | Rabbit |
| Pdk1 | Cell Signaling Technology | #3820 | Rabbit |
| Pfk1 | Santa Cruz | sc-377346 | Mouse |
| HIF-1α | Cell Signaling Technology | #36169 | Rabbit |
| RAP1 | Cell Biolabs | STA-406-1 | Goat |

**Table S3. Chemical synthesis of TCG-biotin**

^^

To a solution of **1** (100 mg, 0.258mmol) and **2** (62.9 mg, 0.258 mmol) in DMF (1 mL) was added EDCI (59 mg, 0.31 mmol) and DMAP (6 mg, 0.048 mmol). The resulting solution was stirred at 25 ℃ for 18 h under argon atmosphere. Water (10 mL) was added to the reacting solution. The organic fraction was separated from the aqueous phase. The aqueous phase was further extracted with DCM (3 x 20 mL). The combined organic layers were washed with brine, dried over Na_2_SO_4_, filtered, and concentrated under reduced pressure. The crude product was further purified by flash columnchromatography, eluting with hexane: EtOAc (5:1 to 2:1, v:v) to afford **3** (87 mg, 0.142 mmol, 55%) as a white solid. ^1^H NMR (500 MHz, Chloroform-*d*) δ 6.93 (d, *J* = 7.9 Hz, 1H), 6.81 – 6.76 (m, 2H), 6.71 – 6.65 (m, 2H), 6.62 (s, 1H), 4.53 (s, 1H), 4.35 (s, 1H), 4.09 – 4.02 (m, 2H), 3.85 (s, 3H), 3.83 (s, 3H), 3.75 (s, 3H), 3.22 – 3.13 (m, 2H), 3.00 – 2.88 (m, 3H), 2.80 – 2.72 (m, 1H), 2.60 – 2.50 (m, 4H), 2.22 (t, *J* = 7.6 Hz, 1H), 2.06 – 1.96 (m, 1H), 1.84 – 1.75 (m, 3H), 1.34 – 1.30 (m, 2H). ^13^C NMR (125 MHz, CDCl_3_) δ 179.02, 171.80, 151.10, 149.19, 147.89, 139.10, 133.69, 131.19, 122.79, 122.67, 121.01, 114.68, 112.33, 111.52, 76.42, 70.50, 62.32, 60.44, 56.12, 56.09, 56.04, 55.55, 44.02, 42.09, 40.67, 33.67, 31.58, 29.84, 28.40, 24.95, 14.26. LRMS (ESI) m/z: 615.2 [M + H]^+^.

^1^H NMR spectrum of compound 3 (TCG-Biotin)


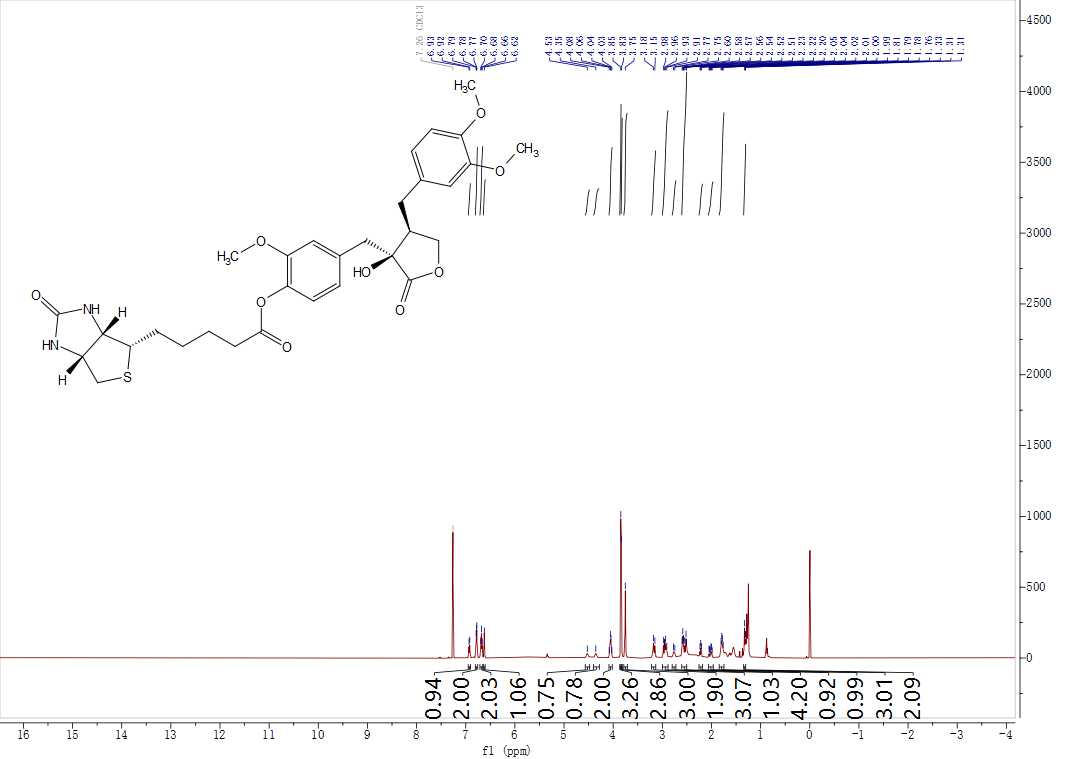


^13^C NMR spectrum of compound 3 (TCG-Biotin)


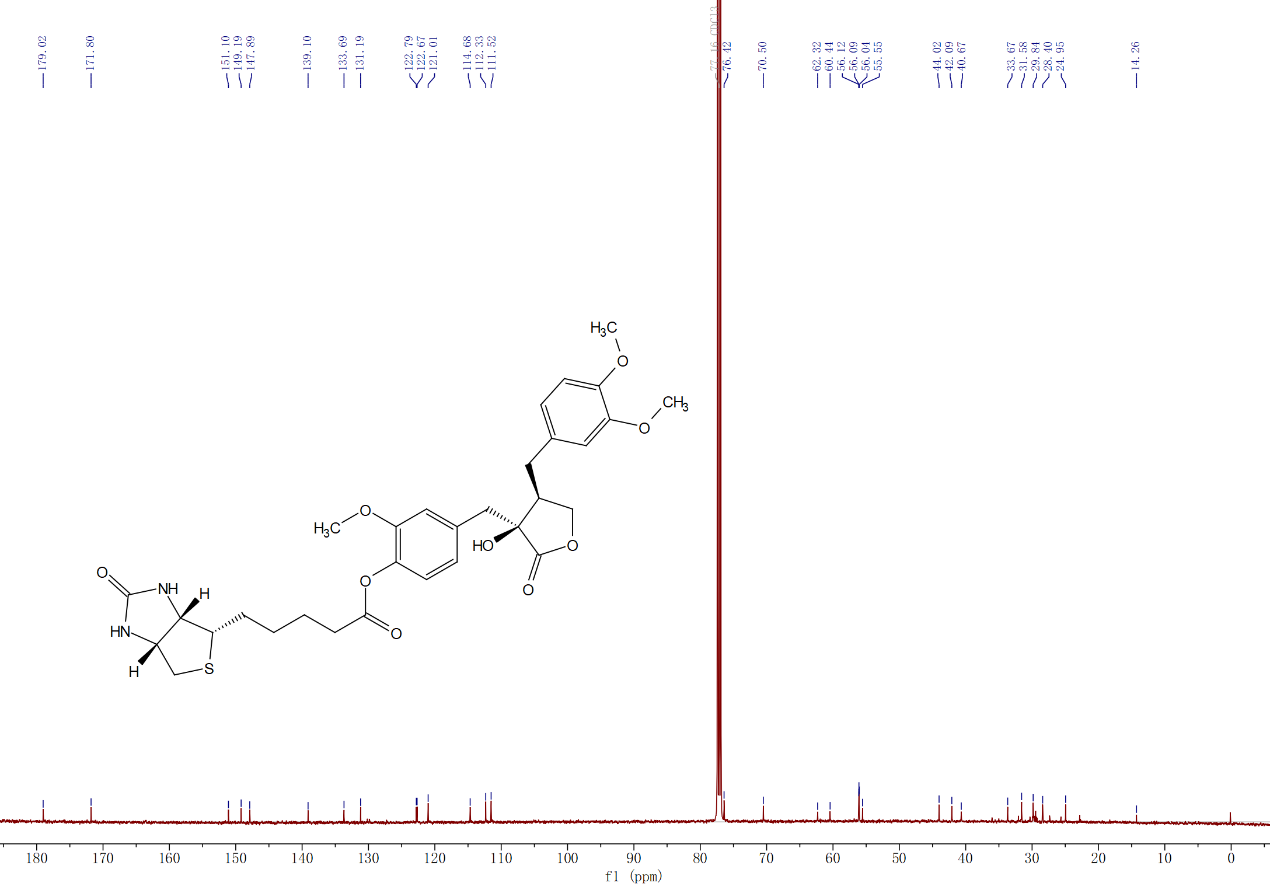


**Table S4. Chemical structure of TCG.**

| Name | Trachelogenin |
| --- | --- |
| Molecular Formula | C_21_H_24_O_7_ |
| Molecular Weight | 388.4 |
| CAS | 34209-69-3 |
| Purity | ≥98% |
